# Seroprevalence of antibodies against SARS-CoV-2 in the adult population during the pre-vaccination period, Norway, winter 2020/21

**DOI:** 10.2807/1560-7917.ES.2022.27.13.2100376

**Published:** 2022-03-31

**Authors:** Erik Eik Anda, Tonje Braaten, Kristin Benjaminsen Borch, Therese Haugdahl Nøst, Sairah L F Chen, Marko Lukic, Eiliv Lund, Frode Forland, David A Leon, Brita Askeland Winje, Anne-Marte Bakken Kran, Mette Kalager, Fridtjof Lund Johansen, Torkjel M Sandanger

**Affiliations:** 1 Department of Community Medicine, UiT The Arctic University of Norway, Tromsø, Norway; 2 Norwegian Institute of Public Health, Oslo, Norway; 3 Department of Non-communicable Disease Epidemiology, London School of Hygiene and Tropical Medicine, London, United Kingdom; 4 Clinical Effectiveness Research Group, University of Oslo, Oslo, Norway; 5 Clinical Effectiveness Research Group, Oslo University Hospital, Oslo, Norway; 6 Department of Immunology, Oslo University Hospital, Rikshospitalet, Oslo, Norway

**Keywords:** Seroprevalence, SARS-CoV-2, Covid-19, Norway, contact tracing, cumulative incidence

## Abstract

**Background:**

Since March 2020, 440 million people worldwide have been diagnosed with COVID-19, but the true number of infections with SARS-CoV-2 is higher. SARS-CoV-2 antibody seroprevalence can add crucial epidemiological information about population infection dynamics.

**Aim:**

To provide a large population-based SARS-CoV-2 seroprevalence survey from Norway; we estimated SARS-CoV-2 seroprevalence before introduction of vaccines and described its distribution across demographic groups.

**Methods:**

In this population-based cross-sectional study, a total of 110,000 people aged 16 years or older were randomly selected during November–December 2020 and invited to complete a questionnaire and provide a dried blood spot (DBS) sample.

**Results:**

The response rate was 30% (31,458/104,637); compliance rate for return of DBS samples was 88% (27,700/31,458). National weighted and adjusted seroprevalence was 0.9% (95% CI (confidence interval): 0.7–1.0). Seroprevalence was highest among those aged 16–19 years (1.9%; 95% CI: 0.9–2.9), those born outside the Nordic countries 1.4% (95% CI: 1.0–1.9), and in the counties of Oslo 1.7% (95% CI: 1.2–2.2) and Vestland 1.4% (95% CI: 0.9–1.8). The ratio of SARS-CoV-2 seroprevalence (0.9%) to cumulative incidence of virologically detected cases by mid-December 2020 (0.8%) was slightly above one. SARS-CoV-2 seroprevalence was low before introduction of vaccines in Norway and was comparable to virologically detected cases, indicating that most cases in the first 10 months of the pandemic were detected.

**Conclusion:**

Findings suggest that preventive measures including contact tracing have been effective, people complied with physical distancing recommendations, and local efforts to contain outbreaks have been essential.

## Introduction

As at 3 March 2022, 440.2 million people worldwide have been diagnosed with coronavirus disease (COVID-19) [1]. However, as these figures are based on the number of virologically detected cases of severe acute respiratory syndrome coronavirus 2 (SARS-CoV-2), they underestimate the true prevalence and incidence of COVID-19 because of limited test coverage, symptom-based test strategies, and the occurrence of asymptomatic cases [2,3]. This underestimation limits our understanding of the spread of SARS-CoV-2 and impedes the development of effective public health strategies. The seroprevalence, i.e. the number of individuals with antibodies present in a defined population at a given time, of antibodies against SARS-CoV-2 can provide useful and needed estimates of the number of people that have been infected [4,5]. Typically, IgG antibodies appear in the blood within 4 weeks of infection with a microbe and thus serve as an indicator of past infection [6]. Although the level of SARS-CoV-2 antibodies is suspected to decline several months after infection [7], the window for antibody detection is longer than that for virus detection.

A large meta-analysis from 2021 [8] reported varied SARS-CoV-2 seroprevalence, from 1.7 and 4.7% in the WHO Regions Western Pacific and Europe, to 19.6% in India. Moreover, the ratio of SARS-CoV-2 seroprevalence to the cumulative incidence of virologically detected cases was 8.4 in the European Region, indicating that for each virologically detected SARS-CoV-2 case, at least eight remained undetected (Spearman's rank correlation coefficient across all locations was 0.59) [8]. In Norway, 44,356 virologically detected SARS-CoV-2 cases had been reported by 20 December 2020, suggesting a cumulative incidence proportion of 0.8% [9]. The Norwegian cumulative incidence numbers indicated a first wave of infections in March 2020, which started before national lockdown, and a second wave from October 2020 to January 2021.

Up to March 2021, no large study with a population-based random sample has estimated SARS-CoV-2 seroprevalence in Norway. Three smaller studies have estimated a seroprevalence of 1.0% (n = 900) and 0.6% (n = 1,812) in Norway, and 1.4% in Oslo (n = 9,765, sampled over a 32-week period) [10-12]. An accurate estimate of seroprevalence in Norway was important in the early phases of the COVID-19 pandemic for containment and vaccination strategies, for estimating infection fatality rates, and for assessing the effectiveness of implemented restrictions or non-pharmaceutical interventions. Experiences from Norway’s low-density population setting may apply to other similar regions and could be valuable in creating strategies to manage COVID-19 going forward, and for future pandemics.

Thus, we aimed to estimate SARS-CoV-2 seroprevalence in a representative sample of inhabitants of Norway before the introduction of vaccines and to describe the distribution of this seroprevalence across relevant demographic groups.

## Methods

### Study population

**Figure 1:**
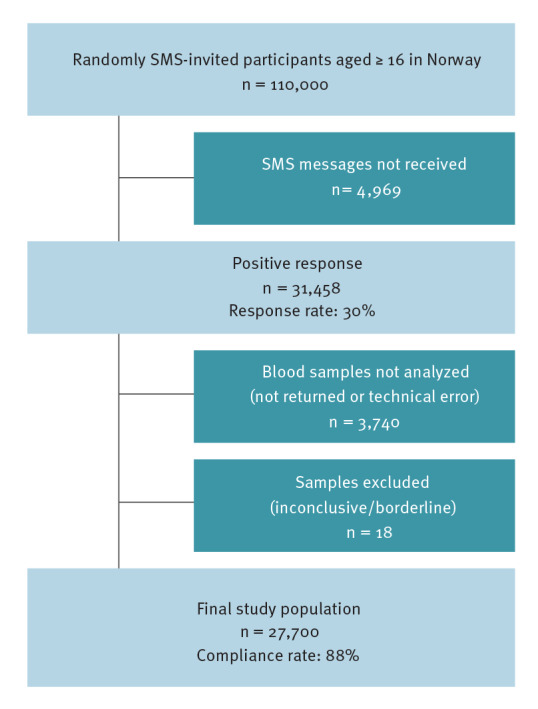
Flowchart of the invited population and final study sample for a SARS-CoV-2 antibody seroprevalence study, Norway, winter 2020/21 (n = 110,000 people invited)

SARS-CoV-2: severe acute respiratory syndrome coronavirus 2; SMS: short message service.

This population-based, cross-sectional study included adults (≥ 16 years) in Norway. Children under 16 were not included for two main reasons: (i) the time needed to obtain permissions for biological samples from children were not feasible with the aim to sample in the fall of 2020 and (ii) if the permissions were available, there was no reliable way of contacting the children for participation. For the same reason related to contact and inclusion, individuals living in prisons, nursing homes, or long-term psychiatric institutions (all of whom represent ca 1% of the national population [13]) were not eligible for inclusion. To be eligible, individuals had to have a national identity number, known country of birth, a registered Norwegian address and a mobile phone number. As previous population-based studies have demonstrated that response rates are not evenly distributed across age groups [14], we used a sampling frame from the Norwegian Institute of Public Health (NIPH), which suggests oversampling of specific age groups, namely 16–19 years (x 2), 20–29 years (x 1.5), 65–74 years (x 1.5), and 75 years and older (x 2); we used the same age groups as NIPH to be able to compare our results directly with national reports. Those born outside the Nordic countries were also oversampled (x 2). Based on these methods, in November–December 2020, a total of 110,000 eligible individuals randomly selected from the National Population Register were invited to participate via short message service (SMS). Of the total, 31,458 indicated their willingness to participate. They were sent information about the study and were asked to complete an electronic or paper questionnaire and donate a dried blood spot (DBS) sample. Of these, 27,700 completed the questionnaire and returned the DBS sample (Figure 1). The DBS samples were asked to be returned via post as soon as possible; we included DBS samples sent to the laboratories until 15 February 2021.

The compliance rate is the proportion of people who received sampling kits who managed to return an analytically acceptable sample.

### Data collection

The questionnaire (Supplement S1: Questionnaire – COVID-19 and Immunity in Norway) collected information on education level, occupation, number of people per household and status of SARS-CoV-2 infection, diagnoses of other diseases and symptoms. A total of 1,505 participants of 31,458 did not complete the questionnaire. Information on age, sex, place of birth (Nordic countries or outside), and county of residence was extracted from Statistics Norway.

DBS samples were collected using a self-sampling kit for antibody testing (Vitas Analytical Services, Oslo, Norway). Information was provided on how to perform DBS sampling correctly, including online video instructions. Participants were to place captured capillary blood, after finger prick with a lancet, onto two circles on a filter paper card, which could collect 50 µL each. Participants then placed the filter paper card in a resealable bag containing desiccant, deposited that bag in a return envelope addressed to Vitas, and sent it by regular mail. DBS samples arrived between 25 November 2020 and 15 February 2021, with the bulk arriving in mid- to late-December. Upon receipt, the samples were extracted as follows: they used a manual or automated hole puncher, punched one 3.1 mm punch into a deep-96-well plate. Then, 150 μl Euroimmun kit sample buffer was added and eluted at 2–8°C overnight. Finally, 100 μl from each well were transferred into the ELISA microtitre plate and processed following the standard ELISA protocol. Vitas then stored samples at −80°C until analysis.

### Detection of SARS-CoV-2 antibodies

Analyses of SARS-CoV-2 IgG antibodies were performed at Vitas, using a Euroimmun (Lübeck, Germany) anti-SARS-CoV-2 assay, an enzyme linked immunosorbent assay (ELISA) that provides semiquantitative in vitro detection of human antibodies against the SARS-CoV-2 spike S1 protein. A test panel consisting of pre-pandemic samples and sera from PCR-confirmed COVID-19 convalescent individuals showed greater than 94% sensitivity and 99.9% specificity, but the sample size for that validation was limited (n = 601). The test diagnostics were established in community-based patients. To minimise false positives, all samples that were positive or borderline by the Euroimmun assay [15] underwent confirmatory analysis at the Department of Immunology at Oslo University Hospital. This consisted of a multiplexed in-house bead-based flow cytometric assay, which analysed antibodies against receptor-binding domain (RBD) and the full-length spike protein. The cut-off was set to obtain a specificity of 99.9%; sensitivity was 84% and 92% when including borderline values. Analytical methods are described in more detail in the Supplement S2: Analytical methods for detection of SARS-CoV-2 antibodies.

### Data treatment and statistical analyses

We defined SARS-CoV-2 IgG seropositivity as a Euroimmun value of above 0.8, an RBD above 5, and a spike value above 5, in addition to a signal for background noise/blank below 3,000 in the multiplexed flow cytometric methods. RBD and spike values correspond to signal values measured for antibodies to RBD and full-length spike protein. The signals correspond to median fluorescence intensity (MFI) from anti-human IgG measured from beads coupled with the respective proteins divided by MFI of beads with no antigen. If all three IgG thresholds were met, the sample was considered positive even if background noise slightly exceeded 3,000. Twelve samples only contained enough blood for the Euroimmun assay. Of these, four were positive, with values above 3.8, the highest observed among samples that were considered negative after flow cytometric analyses. We excluded samples with undetermined status (because of elevated blanks and below-threshold values in confirmatory analyses, or borderline Euroimmun results with too little blood for confirmatory analyses; n = 18 samples).

We dichotomised IgG antibody presence (yes/no) in our statistical analysis. Seroprevalence was estimated for the total study sample, and by age group (16–19, 20–44, 45–66, 67–79, ≥ 80 years), sex (women/men), place of birth (Nordic or outside Nordic countries), county (n = 11), educational level (primary school/junior high school, high school, vocational school, university or college), occupation (healthcare worker: yes/no), and number of people living in the household (1, 2–4, or ≥ 5). Given that we received samples throughout February 2021, which was in the second wave of the pandemic in Norway, we wanted to evaluate the temporal stability of our estimate by comparing rates before and after 1 January 2021. Overall and subgroup seroprevalence was calculated by the number of seropositive individuals divided by the number of individuals who returned DBS samples. Seroprevalence is presented as percentages with 95% confidence intervals (CI). We calculated the ratio of SARS-CoV-2 seroprevalence to the cumulative incidence of virologically detected cases registered at the Norwegian Institute of Public Health [9] by 20 December 2020. We were not able to exclude data on individuals younger than 16 years of age from virologically detected cases.

We used rake weighting to adjust population estimates of seroprevalence by age, sex, place of birth (Nordic or outside Nordic countries), and county based on individual-level data for the invited sample (participants and non-responders) together with the corresponding distributions from the source population, provided by the Norwegian Population Register. We applied propensity scores for nonresponse adjustment and jackknife replicate weights for the raking procedure [16]. The estimates were subsequently corrected for test performance [17]. We also retrieved population-level data on age, sex, place of birth, county of residence, education level, and occupation (healthcare worker: yes/no) registered in Statistics Norway as at 1 January 2021. Correlation between weighted seroprevalence and cumulative incidence in the different counties at the time when most DBS samples were sent to the laboratory was calculated using Pearson’s correlation coefficient. All data treatment and statistical analyses were performed using STATA, release 16 (Stata corp, College Station, Texas, United States).

### Ethical statement

All participants gave written or electronic informed consent to participate in the study. The project group adheres to the Helsinki Declaration. This study has been approved by the Regional Committee for Medical Research Ethics, North Norway (reference number: 154985/2020) and the Norwegian Data Protection Authority (reference number: 758042/2020).

## Results

Mean age in the study sample was 49.4 years (SD: 17.3). Participants aged 20–44 years comprised 36.8% of the sample, 40.5% were aged 45–66 years, 3.1% were aged younger than 20 years, and 2.8% were 80 years or older. More women (57.4%) than men (42.6%) participated, and 86.9% were born in Nordic countries. The distribution of participants by county was close to the national average. More than half of participants (52.5%) reported a university or college education level, and most (74.5%) had 2–4 people living in their household. Single-person households represented 16.7%, and those with five or more people represented 8.8%; 14.2% of participants reported that they were healthcare workers (Table 1).

**Table 1:** Descriptive summaries of the SARS-CoV-2 antibody seroprevalence study sample, non-responders and national population statistics, Norway, winter 2020/21 (n = 104,637)

Variables	Study sample n = 27,700		Non-responders n = 76,937		National^a^
	%	n	%	n	%
**Data from Statistics Norway**					
Age					
Mean (SD)	49.4	17.3	47.5	20.2	NA
Age groups					
16–19	3.1	868	6.3	4,834	5.7
20–44	36.8	10,190	41.8	32,186	40.7
45–66	40.5	11,206	30.2	23,202	34.3
67–79	16.8	4,655	14.6	11,254	13.9
≥ 80	2.8	781	7.1	5,461	5.4
Sex					
Women	57.4	15,895	47.8	36,798	49.6
Men	42.6	11,793	52.2	40,134	50.4
Place of birth					
Nordic countries	86.9	24,062	71.5	54,976	82.5
Outside Nordic countries	13.1	3,626	28.5	21,956	17.5
Counties					
Troms and Finnmark	6.1	1,685	4.3	3,340	4.5
Nordland	4.4	1,213	4.6	3,498	4.5
Trøndelag	8.4	2,314	8.7	6,687	8.7
Møre and Romsdal	4.5	1,256	5.1	3,908	4.9
Vestland	12.0	3,326	11.4	8,777	11.8
Rogaland	7.5	2,072	8.4	6,479	9.0
Agder	4.9	1,362	5.9	4,524	5.7
Vestfold and Telemark	7.8	2,155	8.0	6,126	7.8
Viken	24.3	6,714	23.4	17,977	23.2
Oslo	13.7	3,782	13.1	10,039	12.9
Innlandet	6.5	1,806	7.3	5,577	6.9
**Data from questionnaire**					
Education level					
Primary school/junior high school	7.7	2,022	NA	NA	25.3
High school	25.9	6,766	NA	NA	37.0
Vocational school	13.8	3,617	NA	NA	3.0
University or college	52.5	13,736	NA	NA	34.6
Healthcare worker					
Yes	14.2	3,716	NA	NA	22.0
No	85.8	22,472	NA	NA	78.0
Number of people living in household					
1	16.7	4,376	NA	NA	39.3
2–4	74.5	19,500	NA	NA	55.0
≥ 5	8.8	2,312	NA	NA	15.7

NA: not applicable; SARS-CoV-2: severe acute respiratory syndrome coronavirus 2; SD: standard deviation.

^a^ Numbers from Statistics Norway [13] for those aged 16 years and older, available only in percentages.

**Figure 2:**
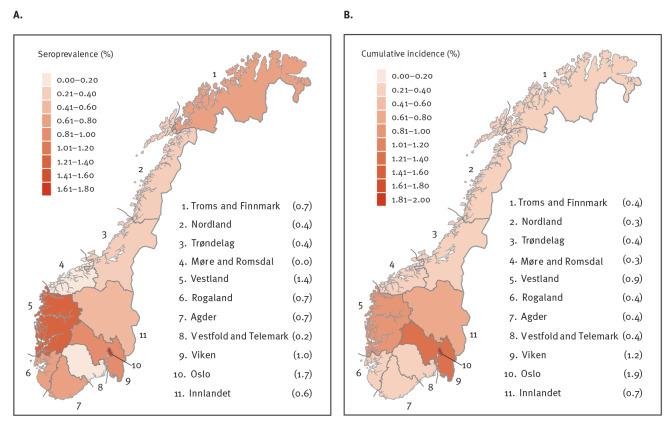
(A) Weighted seroprevalence (n = 234) and (B) cumulative incidence (n = 44,404) of SARS-CoV-2 infection by county, Norway, December 2020

By 20 December 2020, 44,356 individuals had been diagnosed with SARS-CoV-2 in Norway, for an estimated COVID-19 cumulative incidence proportion of 0.8% [9] (Figure 2). The weighted and adjusted SARS-CoV-2 seroprevalence was 0.9% (95% CI: 0.7–1.0) based on a total of 234 seropositive cases (Table 2). The crude and weighted estimates were similar in different subgroups, with the highest seroprevalence observed in those aged 16–19 years (1.9%; 95% CI: 0.9–2.9), among Norwegians born outside the Nordic countries (1.4%; 95% CI: 1.0–1.9), and in the counties of Oslo (1.7%; 95% CI: 1.2–2.2) and Vestland (1.4%; 95% CI: 0.9–1.8). The lowest seroprevalence was observed in the county of Møre and Romsdal (0%; 95% CI: 0.0–0.4). The seroprevalence among healthcare workers (1.1%; 95% CI: 0.8–1.5) was only marginally higher than for those in other professions (0.8%; 95% CI: 0.7–0.9). Sex, education level, number of people living in the household, and occupation showed minor differences in seroprevalence between groups. 

SARS-CoV-2: severe acute respiratory syndrome coronavirus 2.

The median sampling period was late December 2020, ranging late November until mid-February. The cumulative incidence was obtained for 20 December 2020. Maps provided by the Norwegian Mapping Authority [9], Hønefoss, Norway, modified by UiT The Arctic University of Norway, Tromsø, Norway.

**Table 2:** SARS-CoV-2 IgG seropositivity in selected demographic groups of the final study sample, Norway, winter 2020/21 (n = 234)

Variables	Positive tests	Crude seroprevalence			Weighted seroprevalence	
	n	%	95% CI		%	95% CI
**Data from Statistics Norway**						
IgG seropositive cases	234	0.8		0.7–1.0	0.9	0.7–1.0
Age groups						
16–19	16	1.8		1.1–3.0	1.9	0.9–2.9
20–44	100	1.0		0.8–1.2	1.0	0.8–1.2
45–66	86	0.8		0.6–1.0	0.7	0.6–0.9
67–79	29	0.6		0.4–0.9	0.5	0.3–0.7
≥ 80	3	0.4		0.1–1.2	0.4	0.0–0.9
Sex						
Women	133	0.8		0.7–1.0	0.8	0.6–1.0
Men	101	0.9		0.7–1.0	0.9	0.7–1.1
Place of birth						
Nordic countries	184	0.8		0.7–0.9	0.7	0.6–0.9
Outside Nordic countries	50	1.4		1.0–1.8	1.4	1.0–1.9
Counties						
Troms and Finnmark	12	0.7		0.4–1.3	0.7	0.2–1.1
Nordland	6	0.5		0.3–1.1	0.4	0.0–0.9
Trøndelag	8	0.4		0.2–0.7	0.4	0.0–0.8
Møre and Romsdal	1	0.1		0.0–0.6	0.0	0.0–0.1
Vestland	39	1.2		0.9–1.6	1.4	0.9–1.8
Rogaland	15	0.7		0.4–1.2	0.7	0.3–1.2
Agder	9	0.7		0.3–1.3	0.7	0.1–1.2
Vestfold and Telemark	6	0.3		0.1–0.6	0.2	0.0–0.4
Viken	67	1.0		0.8–1.3	1.0	0.7–1.3
Oslo	60	1.6		1.2–2.0	1.7	1.2–2.2
Innlandet	10	0.6		0.3–1.0	0.6	0.2–1.1
**Data from questionnaire**						
Education level						
Primary school/junior high school	17	0.8		0.5–1.3	NA	
High school	54	0.8		0.6–1.0	NA	
Vocational school	22	0.6		0.4–0.9	NA	
University or college	127	0.9		0.8–1.1	NA	
Healthcare worker						
Yes	42	1.1		0.8–1.5	NA	
No	178	0.8		0.7–0.9	NA	
Number of people living in household						
1	37	0.8		0.6–1.2	NA	
2–4	161	0.8		0.7–1.0	NA	
≥ 5	22	1.0		0.6–1.4	NA	

CI: 95% confidence interval; NA: not applicable; SARS-CoV-2: severe acute respiratory syndrome coronavirus 2.

The maps in Figures 2A and 2B show the spatial variation in weighted seroprevalence across the counties of Norway and the cumulative incidence of virologically confirmed cases in the same counties as at 20 December 2020. The ratio of seroprevalence to the cumulative incidence of virologically detected cases was 1.1 and the Pearson’s correlation coefficient between weighted seroprevalence and cumulative incidence per county was r = 0.84. The estimated seroprevalence was 0.7% (95% CI: 0.6–0.9) before 1 January 2021 and 1.0% (95% CI: 0.9–1.2) after 1 January 2021. Recruitment was completed by December 2020, but the sampling campaign had to be extended until February 2021 because of delays in packaging and shipments during Christmas holidays.

## Discussion

In this random-sample, population-based study, we found a low SARS-CoV-2 seroprevalence (0.9%) in the Norwegian population by January 2021. Seroprevalence was highest in those aged 16–19 years (1.9%), in Oslo County (1.7%), and among individuals born outside the Nordic countries (1.4%). There were considerable geographical differences, with the lowest seroprevalence observed in Møre and Romsdal County (0%). Finally, we found a low ratio (1.1) of seroprevalence to the cumulative incidence of virologically detected cases, indicating that a substantial number of the COVID-19 cases in Norway are detected [8].

Our observed national seroprevalence (0.9%) is supported by two studies performed in residual clinical samples from hospital laboratories in Norway, which reported national seroprevalences of 1.0% in spring [11] and 0.6% in early autumn [10] of 2020. A low national seroprevalence is thus in agreement with other studies performed in 2020 and is likely attributed to the rapid nationwide lockdown and the effective implementation of testing, isolation, contact tracing and quarantine coordinated by local health authorities. On a national level, the lockdown included closed national borders, closed schools and nurseries as well as prohibiting cultural and sporting events. In the fall of 2020, restrictions were coordinated at a more local level, and this survey was completed during this period and amidst the second wave of infections. Based on the low seroprevalence detected, it seems evident that the Norwegian population – to a large extent –has complied with the ongoing regulations and recommendations. We saw substantial geographical differences, with the highest seroprevalences observed in counties with the two largest cities in Norway, Oslo and Bergen, and previous seroprevalence studies from Oslo support our results [18]. The airborne and close-contact nature of SARS-CoV-2 transmission, factors related to high population density in urban settings, such as frequent human interactions and a high number of national and international flights, as well as younger population distributions, may explain these results. Moreover, the key public health strategy of testing, isolation, contact tracing and quarantine is more challenging in large cities than in smaller communities.

Seroprevalence was highest in the youngest age group, a trend that was also found in the United Kingdom (UK), where the highest seroprevalence was reported in those aged 18–24 years (7.9%) [19]. We speculate that young adults have more frequent contact with other individuals, and in larger groups, thus facilitating infection. This contact could be attributable to school, greater social needs, or the higher concentrations of young people living in Norway’s urban centres, which likely increases the risk of SARS-CoV-2 infection, regardless of age. On average, young adults infected with SARS-CoV-2 experience milder symptoms than adults above 40 years of age [20], which may result in a lower motivation to be tested and/or to reduce social contact.

We observed a higher seroprevalence among persons born outside Nordic countries (1.4%). This group is over-represented in Norway’s larger cities, indicating a possible contributing role of population density in driving higher seroprevalence. However, we may not have achieved national representativeness for this group, as the proportion of individuals in our study sample with higher education and born outside of the Nordic countries was greater than in the general population [13]. Language barriers and scepticism towards the SMS invitation are plausible reasons for not capturing a representative sample of this group.

In contrast to the higher seroprevalence observed among healthcare workers in many other countries [8], seroprevalences were similar in healthcare and non-healthcare workers in our study. Low infection rates, few hospitalised patients (total n = 2,623 up to 15 February 2021 [9]) and good access to personal protective equipment likely explain our result. There may be a subtle difference between the seroprevalences of healthcare workers in high-risk settings compared with other healthcare workers, but our study did not differentiate between these groups. In other settings, IgG antibodies were present in 91% of Belgian healthcare workers 168.5 days post infection (median period) [21] and present in greater than 70% of COVID-19 convalescent plasma donors 12 months after infection in Wuhan, China [22]. The latter study reports that 5.4% of the participants did not display detectable IgG levels (above cut-off). While lacking antibodies could be attributed to low test-sensitivity [22], convalescent individuals showing a mild or asymptomatic disease progression could also have undetectable antibody levels.

Differences in education level did not appear to affect seroprevalence. This variable is highly age-dependent, as lower age usually implies lower completed education level. Despite this, the lower education group in our study did not have higher seroprevalence. Lastly, we found no statistically significant differences in seroprevalence between those living alone and those living in larger households, in contrast to earlier findings [19].

When comparing the Norwegian seroprevalence of 0.9% to those of the serological studies assessed in the recent review by Chen et. al. [8], it is evident that Norway has one of the lowest seroprevalences globally. The estimated pooled seroprevalence in the world general population as of 22 December 2020, was 8.0% (95% CI: 6.8–9.2), with the lowest observed in the Western Pacific region (1.7%; 95% CI: 0.0–5.0), and an average of 4.7% (95% CI: 3.6–5.9) in the European Region [8]. In our study and in a UK sample, the highest seroprevalence was found in the youngest age group [19]. However, in the review by Chen et al. [8], this age group was reported to have the lowest seroprevalence globally (2.1%). Whether this is a coincidence or reflects similar infection rates globally in this age group is unknown.

Both the correlation (r = 0.84) between and the ratio (1.1) of seroprevalence to the cumulative incidence confirm the low incidence of SARS-Cov-2 in Norway. Globally, none of the countries included in the Chen et. al., meta-analysis [8] had a ratio lower than or equal to 1 and the European average was 8.4. The lowest ratio was found in the state of Utah in the United States (2.4), and the highest ratio was found in India (56.5) [8], likely due to variations in testing capacities and opportunities in the different countries. In addition, the regression coefficient describing the relationship between seroprevalence and reported cases stratified by county is high enough to indicate low numbers of undetected cases in Norway. The low seroprevalence in Norway is believed to be mainly explained by adherence to national and local restrictions described above and the many sparsely populated regions in Norway. The national guidelines are believed to explain any differences in seroprevalences to neighbouring countries.

Compared to the peak of registered new cases during winter 2021/22 (29,470 new cases at 7 February 2022), the peak during winter 2020/21 was small (938 new cases reported at 4 January 2021, although testing regimes and reporting varied somewhat) [23]. Our study revealed that SARS-CoV-2 seroprevalence in Norway was low during winter 2020/21, before vaccines were introduced. Furthermore, our findings suggest that the proportion of virologically undetected cases was low. The low seroprevalence left Norway particularly vulnerable to a third wave during the spring of 2021, as there was no indication of past infection on a population level that might confer protection. Norway did indeed experience the largest wave thus far from early January until early May 2021. However, the country managed to keep infection rates and hospitalisations relatively low by imposing continuous restrictions. Many restrictions were lifted in September 2021 after most of the adult population (83% by 15 September) were vaccinated. New infections shifted more towards the younger unvaccinated population, lasting through February 2022. Even with the highest daily infection rates peaking in February 2022, COVID-19-related intensive care patients declined. Considering the low Norwegian seroprevalence status at the beginning of 2021, our findings show that it was the correct decision to slow down the infection rates by imposing restrictions until the population was vaccinated and/or the virus became less severe. 

### Strengths and limitations

The strengths of our study include the random sample, population-based study design, high completion rate of DBS samples (88%; 27,700/31,458), and the low risk of overestimation of seroprevalence. There are no stability issues related to the time elapsed from DBS sampling to time of arrival at the laboratory. Generally, DBS is thought to be less accurate than using serum and in a comparison study from May 2021 [24], they concluded a 96.8% accuracy for positive agreement and 81.3% for negative agreement. Given the circumstances, screening a population in a low seroprevalence setting, our main concern was the positive predictive value (PPV). We are confident that the specificity and double-testing regime in this study supplied a reliable and sufficiently high PPV. However, this study also has limitations. The response rate was 30% (31,458/104,637). However, the age and sex distributions were similar to that of the Norwegian general population, so the overall results may still be representative of the population. However, even though we oversampled, the results from some subgroups, e.g. those without mobile phones and those lacking a national identity number, may be more uncertain and not representative of the Norwegian population.

Studies involving personal information linked to risk behaviour tend to have a lower response from high-risk groups [25]. However, having antibodies is not necessarily linked to a stigma or viewed as high risk. Because of the low response rate in the youngest age group and because this group had the highest seroprevalence, the estimates could be too low. On the other hand, weighting and adjustments for sensitivity and specificity of results reduced under-reporting to a minimum.

There are several reasons why seroprevalence may be underestimated in our study. First, those who were very recently infected were less likely to have detectable antibody levels at the time of sampling [26]. Second, because of the sensitivity of the two tests (94% and 84%), some false negative results were expected, but because of the low prevalence in general, these cases are few. Furthermore, the properties of the tests used to detect antibodies might differ between populations with a different case-mix [27]. It is possible that in a region/population in which the proportion of asymptomatic cases was high, the tests would perform with somewhat lower sensitivity, resulting in an underestimated seroprevalence in that specific population. The proportion of participants with only primary school/junior high school was low in our study population. We did not test children under the age of 16 years or individuals in nursing homes. Calculations based on the available numbers for cumulative incidence in these two groups, i.e. those aged 16 and older (0.8%; 7,246/948,847) and nursing home residents (3%; 862/28,500) [28] and the number of potential participants from the same groups in our study, this would not have impacted the overall seroprevalence significantly as long as these two groups display similar correlations between seroprevalence and cumulative incidence as the other age groups. This limitation is only relevant when comparing seroprevalence with cumulative incidence, not when estimating seroprevalence in the adult population.

## Conclusion

Although there are limitations to seroprevalence estimates, such as time between infection and antibody testing (waning antibodies over time or testing before antibodies develop) and individual antibody response to the infection, IgG antibody seroprevalence is probably the best indication of population protection and past infection, irrespective of SARS-CoV-2 test capacity or availability in the population.

## Supplementary Data

364000 Supplement
